# Incidence of *Plasmodium falciparum* malaria infection in 6-month to 45-year-olds on selected areas of Bioko Island, Equatorial Guinea

**DOI:** 10.1186/s12936-021-03850-8

**Published:** 2021-07-20

**Authors:** Vicente Urbano Nsue Ndong Nchama, Ali Hamad Said, Ali Mtoro, Gertrudis Owono Bidjimi, Marta Alene Owono, Escolastica Raquel Mansogo Maye, Martin Eka Ondo Mangue, Genaro Nsue Nguema Okomo, Beltran Ekua Ntutumu Pasialo, Dolores Mbang Ondo, Maria-Silvia Angue Lopez, Fortunata Lobede Mochomuemue, Mariano Obiang Obono, Juan Carlos Momo Besaha, Raul Chuquiyauri, Said Abdallah Jongo, Kassim Kamaka, Ummi Abdul Kibondo, Thabit Athuman, Carlos Cortez Falla, Jeremías Nzamio Mba Eyono, Jordan Michael Smith, Guillermo A. García, José Raso, Elizabeth Nyakarungu, Maxmillian Mpina, Tobias Schindler, Claudia Daubenberger, Laurence Lemiale, Peter F. Billingsley, B. Kim Lee Sim, Thomas L. Richie, L. W. Preston Church, Ally Olotu, Marcel Tanner, Stephen L. Hoffman, Salim Abdulla

**Affiliations:** 1 Ministry of Health and Social Welfare, Equatorial Guinea (EGMOHSW), Malabo, Equatorial Guinea; 2 Medical Care Development International (MCDI), Silver Spring, USA; 3 Ifakara Health Institute, Dar es Salaam, Tanzania; 4 Sanaria Inc., Rockville, USA; 5 Swiss Tropical and Public Health Institute (Swiss TPH), Basel, Switzerland; 6 University of Basel, Basel, Switzerland

**Keywords:** Malaria, *Plasmodium falciparum*, Incidence, PfSPZ Vaccine, Malabo, Bioko Island, Equatorial Guinea

## Abstract

**Background:**

Extensive malaria control measures have been implemented on Bioko Island, Equatorial Guinea over the past 16 years, reducing parasite prevalence and malaria-related morbidity and mortality, but without achieving elimination. Malaria vaccines offer hope for reducing the burden to zero. Three phase 1/2 studies have been conducted successfully on Bioko Island to evaluate the safety and efficacy of whole *Plasmodium falciparum* (Pf) sporozoite (SPZ) malaria vaccines. A large, pivotal trial of the safety and efficacy of the radiation-attenuated Sanaria^®^ PfSPZ Vaccine against *P. falciparum* is planned for 2022. This study assessed the incidence of malaria at the phase 3 study site and characterized the influence of socio-demographic factors on the burden of malaria to guide trial design.

**Methods:**

A cohort of 240 randomly selected individuals aged 6 months to 45 years from selected areas of North Bioko Province, Bioko Island, was followed for 24 weeks after clearance of parasitaemia. Assessment of clinical presentation consistent with malaria and thick blood smears were performed every 2 weeks. Incidence of first and multiple malaria infections per person-time of follow-up was estimated, compared between age groups, and examined for associated socio-demographic risk factors.

**Results:**

There were 58 malaria infection episodes observed during the follow up period, including 47 first and 11 repeat infections. The incidence of malaria was 0.25 [95% CI (0.19, 0.32)] and of first malaria was 0.23 [95% CI (0.17, 0.30)] per person per 24 weeks (0.22 in 6–59-month-olds, 0.26 in 5–17-year-olds, 0.20 in 18–45-year-olds). Incidence of first malaria with symptoms was 0.13 [95% CI (0.09, 0.19)] per person per 24 weeks (0.16 in 6–59-month-olds, 0.10 in 5–17-year-olds, 0.11 in 18–45-year-olds). Multivariate assessment showed that study area, gender, malaria positivity at screening, and household socioeconomic status independently predicted the observed incidence of malaria.

**Conclusion:**

Despite intensive malaria control efforts on Bioko Island, local transmission remains and is spread evenly throughout age groups. These incidence rates indicate moderate malaria transmission which may be sufficient to support future larger trials of PfSPZ Vaccine. The long-term goal is to conduct mass vaccination programmes to halt transmission and eliminate *P. falciparum* malaria.

## Background

The burden of malaria is concentrated in sub-Saharan Africa, where 94% of the estimated 229 million malaria cases occurred in 2019, the large majority caused by *Plasmodium falciparum* (Pf). Children and pregnant women are the most vulnerable for dying of the disease, and progress with burden reduction has plateaued [1]. The World Health Organization (WHO) is currently promoting the “high burden to high impact” approach prioritizing eliminating malaria deaths as an immediate, focused response [2]. However, the WHO and multiple international stakeholders have renewed their commitment to long-term malaria control and elimination, and emphasized the need for new tools for tackling the disease. New vector control measures, drugs, and vaccines are under development to control and eliminate malaria, including subunit and whole PfSPZ Vaccines [3].

The Government of Equatorial Guinea (EG) is partnering with Medical Care Development International (MCDI), Ifakara Health Institute (IHI), Swiss Tropical and Public Health Institute (Swiss TPH), and Sanaria Inc. to evaluate the safety and efficacy of the whole *P. falciparum* sporozoite (PfSPZ) malaria vaccine approach, with radiation-attenuated Sanaria^®^ PfSPZ Vaccine and the chemo-attenuated Sanaria^®^ PfSPZ-CVac, the two leading products under development [4, 5]. PfSPZ Vaccines are intended to be offered to whole populations through mass vaccination programmes (MVPs) to eliminate malaria in defined geographic areas.

Three phase 1/2 studies of PfSPZ Vaccines have been conducted in EG. The first study was the first clinical trial in the history of EG and evaluated the safety and tolerability of PfSPZ Vaccine in adults [6]. The second study assessed the safety and tolerability of PfSPZ Vaccine in 6 month- to 65-year-olds and compared the safety, tolerability, and vaccine efficacy (VE) against controlled human malaria infection (CHMI) of PfSPZ Vaccine and PfSPZ-CVac [7]. The third study assessed the safety, tolerability, and VE against CHMI of four different dosage regimens of PfSPZ Vaccine (Jongo SA et al., pers. commun.). These studies and the experience from other studies in Africa, Europe, and the USA have encouraged further development of PfSPZ Vaccines. Planning is underway to conduct a larger randomized, double-blind, placebo-controlled trial of the lead product, PfSPZ Vaccine, to assess the safety and VE against natural exposure to malaria in 2- to 50-year-olds (1400 vaccinees and 700 controls), split into two age groups, 2–12 years and 13–50 years.

An efficacious pre-erythrocytic vaccine, like PfSPZ Vaccine, which interrupts malaria transmission since the development of asexual blood stages and associated sexual stages is prevented [8]. Therefore, the planned trial of PfSPZ Vaccine will focus on VE against the incidence of malaria parasitaemia as detected by thick blood smear (TBS) in 2- to 50-year-olds in selected areas of Bioko Island, EG as the primary efficacy objective. Combined active (biweekly) and passive surveillance will be used to identify incident infections, reading ~ 0.54 µL of blood from a TBS and scoring the TBS as positive or negative. VE against malaria with symptoms (clinical malaria) will also be an objective.

Information on the expected incidence of malaria and malaria with symptoms on Bioko Island is needed to properly design the upcoming trial, including estimating the appropriate sample size [9, 10]. Hence, this study, called EGMALEP (Equatorial Guinea Malaria Epidemiology Project), was conducted to assess the incidence of malaria (parasitaemia) and malaria with symptoms in a closely followed cohort of participants from areas with relatively high malaria prevalence, in and around Malabo. This study used the same outcome variable as will be used in the upcoming vaccine trial—new malaria infection identified by TBS through active and passive surveillance. In addition to measuring incidence, the study assessed the concomitant illnesses that occurred in the study population and piloted tools and procedures, including the active and passive surveillance methods.

## Methods

### Study population

**Fig. 1:**
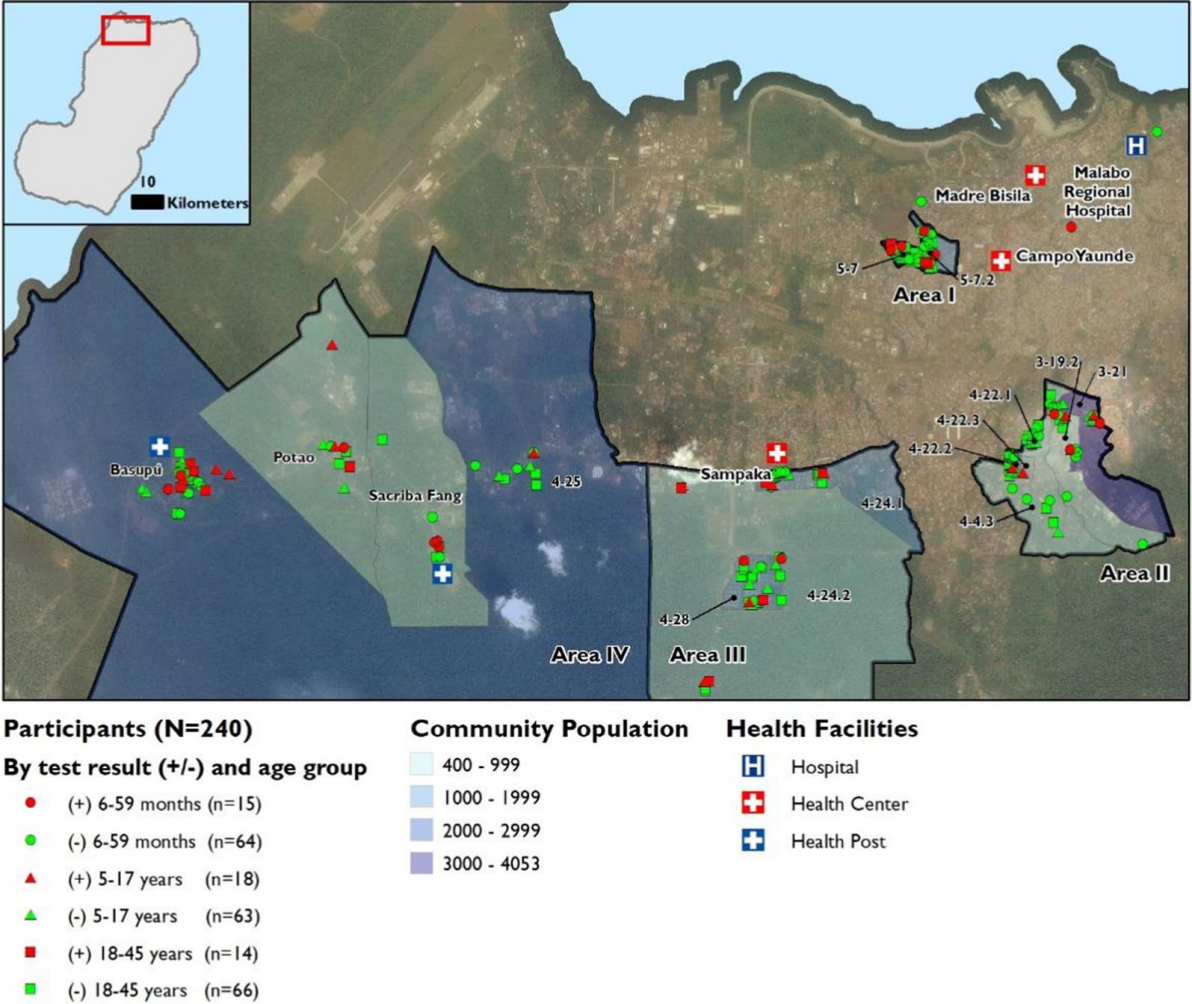
Map of the study areas in the northwest sector of Bioko Island showing population estimates for each community and distribution of study participants showing the place of residence, age group, and whether parasitaemia positive or negative during surveillance

The study was conducted in communities in an urban/peri-urban area of Malabo District in the Bioko North Province on Bioko Island, EG, where the capital city, Malabo, is located. The population of Malabo District is estimated to be 191,671 individuals living in 68,306 households with an average household size of 2.8 individuals (2018 BIMCP unpublished health census data). The population is composed of several ethnic groups, including the Bubi, Fang, Annobones, Ndowe, Bisio, and Fernandino. The study area (Fig. 1) includes a health facility in Sampaka and two primary health posts in Sacriba and Basupú communities. Serious illnesses in these areas are generally cared for at the Malabo Regional Hospital, a government-owned public hospital. There are also two private hospitals in close distance to these communities.

### Malaria on Bioko Island

Malaria has been a major public health problem in EG, and malaria transmission occurs throughout the year, with more cases occurring during and after the rainy season (March–December) [11]. The main malaria species, *P. falciparum*, constitutes over 95% of cases, although *Plasmodium malariae, Plasmodium ovale,* and *Plasmodium vivax* circulate on Bioko Island with low prevalence [12–15].

The major vectors maintaining residual transmission on Bioko are *Anopheles melas* and *Anopheles coluzzii* [16]. Significant reduction of malaria has been achieved on Bioko Island in the last 16 years following intensive malaria control efforts [17]. Since 2004, the Bioko Island Malaria Control Project (BIMCP) and the National Malaria Control Programme (NMCP) have implemented repeated rounds of indoor residual spraying (IRS), mass distribution of long-lasting insecticidal nets (LLINs), larval source management, behaviour change communication activities, and improved case management resulting in 63% reduction in malaria prevalence in 2–14-year-olds in 2019 [11]. Despite these efforts, pockets of high malaria prevalence remain throughout the island, and visitors import additional cases from mainland EG where transmission is more intense [11, 18].

For this study, communities with high malaria prevalence (parasitaemia above 15% by rapid diagnostic test (RDT)) based on a 3-year average (2016–2018) of data from the annual cross-sectional malaria indicator survey were selected. The selected communities were mainly from the western and southwestern regions of the Malabo District; they were categorized based on the administrative boundaries and geolocation into four study areas (area I–IV) (Fig. 1). The study was conducted out of the centrally located Sampaka Health Center (SHC) (Fig. 1).

### Study design and procedures

The EGMALEP was a prospective cohort study conducted from January 28th to September 12nd, 2019. The primary objective was to estimate malaria incidence diagnosed using TBS in the areas planned for the future large trial of PfSPZ Vaccine following clearance of any existing parasitaemias. The secondary objective was to describe the characteristics and clinical presentation of the incident infections as well as assess the performance of RDT. Participants were followed for 24 weeks with biweekly TBSs (active surveillance) augmented by assessing any acute febrile illnesses (passive surveillance). Information on co-morbidities that occurred was collected from participants during study visits through updates on medical history, physical examination, and laboratory investigations. The study targeted the enrollment of 240 participants split evenly into three age groups: 6–59 months, 5–17 years, and 18–45 years.

### Community sensitization and LLIN distribution

Permission was obtained from local government officials and community representatives to use the household database from the BIMCP/NMCP to identify household members to be invited to participate in the study [19]. Subsequently, community meetings were held in the study areas to sensitize the population about the study. LLINs were distributed in the study areas by the BIMCP/NMCP before the start of the study.

### Participant selection

A multistage sampling was done, first communities were selected primarily due to relatively high parasite prevalence in non-travelling children aged 2–14 years and proximity to the Sampaka Health Centre (no more than 6 kms from the facility) based on the BIMCP/NMCP 2018 health census. Individual demographic information from the 15 selected communities, was aggregated to the household level to determine the number of eligible individuals per age group permanently residing in inhabited households within the study area. Households with at least one eligible individual were included in the sampling frame, representing 20,240 eligible individuals residing in 7901 eligible households.

Thereafter, Python 2.7, PANDAS package was used to randomly select and order individuals from unique households for each of the three study age groups. The number of individuals to sample within each of the communities was pre-determined to ensure a representative geographic distribution of sampled individuals proportionate to community sizes. Once an age group was filled, individuals were only invited to participate if contributing to the unfilled age groups.

### Eligibility and enrolment

Potential adult participants, or the parents or guardians of potential pediatric participants, present during household visits were asked to come to SHC to provide written consent, 9–17-year-olds were asked to provide written assent, and 6–8-year-olds were asked to provide verbal assent. Screening evaluation included the history of previous illnesses, and physical examination included vital signs and anthropometric measurements. Blood samples were collected for analysis of malaria parasitaemia (TBS, RDT and quantitative PCR (qPCR), the latter performed retrospectively), complete blood count with differential, and biochemistry including alanine aminotransferase (ALT), aspartate aminotransferase (AST), total bilirubin, creatinine, and glucose. A urine sample was collected for human chorionic gonadotropin pregnancy testing in women aged 9–45 years. Socio-demographic data were also collected.

Participants were considered eligible for the study if they were permanent residents of the study area, age between 6 months and 45 years, and provided consent/assent. Participants were excluded if they had a history or clinical manifestations of serious or chronic disease requiring frequent medical care including human immunodeficiency virus (HIV) infection, clinical tuberculosis, sickle cell disease, malignancies, diabetes mellitus, hypertension, mental illness, or seizures. Additional exclusion criteria were mid-upper arm circumference (MUAC) < 11.5 cm for children under 5 years of age (indicating malnourished state), grade 3 abnormal laboratory parameters (based on local normal range and toxicity grades for haematology and biochemistry), pregnancy, and history of allergy or serious adverse reaction to artemether/lumefantrine (AL). Participants with issues that increased the risk of non-adherence to study procedures, including the intention to move from the study area during the study period, were excluded.

Eligible participants were given a directly observed curative 3-day, six-dose course of artemether–lumefantrine (AL) (Lumet 80^®^ Cipla Ltd, India) according to manufacturer’s instructions [20]. All the AL doses were directly observed by clinical staff or community health care workers. To ensure compliance with taking all doses at home, the prescribed medications were maintained at the clinic pharmacy and single doses were then collected by the community health workers, were taken to participants and were administered under supervision for each specific dosing time point. The participants were rechecked for parasitaemia 14 days later and those who were negative by blood slide were officially enrolled.

### Active surveillance

The enrolled participants were actively followed up at home every 2 weeks for 24 weeks except the 12th and 24th week time points when they were invited for medical evaluation at the SHC. During the home visits, axillary temperature was measured and the participants were asked about their well-being, history of fever in the prior 24 h and travel history in the preceding 2 weeks, and the use of ITNs, IRS and mosquito repellent. If clinically well, a blood sample for TBS, RDT and qPCR was collected by venipuncture. If symptomatic with fever or history of fever, the participant was transported to study clinic for medical evaluation. At the clinic, a medical history, travel history and malaria control intervention history were taken, a clinical examination performed, and the same malaria diagnostic blood sample obtained. All blood samples for TBS, RDT and PCR were collected using ethylene diamine tetra acetic acid (EDTA) tubes and kept in a cooler box to maintain ambient indoor temperature until reaching the laboratory.

Participants were treated based on TBS results. For those TBS positive participants who were at the clinic, treatment with an anti-malarial was initiated before they were allowed to go home. For those who were seen at home they were invited back to the clinic by the community health workers and were then reviewed again at the clinic and an anti-malarial treatment initiated.

Compliance with study visits and procedures was encouraged by the provision of a study calendar to participants, by using multiple contact methods (phone calls to participants/parents/guardians or close contacts provided on enrollment to remind participants of upcoming home or clinic visits), by home visits by community mobilizers, and by provision of transportation to the study center. Participants were compensated for time spent during scheduled study related clinic visits (approximately 12 United States dollars), but not for home visits.

### Passive surveillance

Study doctors were available to see study participants 24 h–7 days a week. Participants were encouraged to visit the SHC or call the study doctors whenever they were sick. Medical history, physical examination and laboratory investigations were done as needed for clinical evaluation. All participants with history of fever had their blood checked for malaria by TBS, RDT and later qPCR.

### Laboratory assessments

Thick and thin blood smears and the RDTs for assessment of malaria parasites were prepared from samples collected during home visits or at the study clinic. These were prepared and independently analysed at the SHC laboratory to allow blinding and standardization of the preparation and reading of the results. All remaining study related laboratory analyses were done at the public health laboratory in Baney which is located 24 km from the SHC. TBSs were prepared and read as described previously [21]. In short, thick and thin smear were made, air dried and then stained with 4% Giemsa for 45 min for samples from asymptomatic participants and 10% Giemsa for 10 min for samples from symptomatic participants. The TBSs were read using a light microscope with a high-power field (immersion oil, 100× objective) of 0.18 mm diameter; 6 passes (0.54 µL of blood) for asymptomatic or 24 passes (2.14 µL of blood) for symptomatic participants, were read before the TBS was declared negative. The slides were read by two independent expert microscopists and any discrepancies were resolved by a third microscopist. Parasite densities were calculated as the number of asexual parasites counted per volume of blood examined. All positive TBSs were verified retrospectively at the public health laboratory in Baney using qPCR, as described previously [15]. In short, the qPCR multiplex assay targeted two independent *Plasmodium* genes namely the Pan-*Plasmodium* 18S RNA sequence (Pspp18S) and the *P. falciparum*-specific acidic terminal sequence of the *var* genes (PfvarATS). The human Ribonuclease P gene (HsRNaseP) was used as a DNA extraction and qPCR amplification control. All qPCR assays were run in duplicate and both non-template control (Molecular grade nuclease-free water) and *P. falciparum* 3D7 DNA were included as negative and positive controls, respectively. The sample was considered positive if the quantitation cycles (Cq) for each of the two replicates was Cq < 40 for *Plasmodium* spp. gene and Cq < 28 for qPCR amplification control. Haematology parameters were established using ABX Pentra 60 C+ (Horiba Medical, USA) and biochemistry parameters using Cobas Integra 400 Plus (Roche, Switzerland). Urine pregnancy tests were done using Hexagon hCG 1-step rapid tests (Human Diagnostics Worldwide, Germany).

### Data management

Information was collected on paper-based study case reports forms and these were entered into a customized electronic database, Castor EDC^R^. Data were entered and verified by independent teams.

### Sample size

The design assumed an incidence of new malaria infections (parasitaemia) over 24 weeks of 10% for each age group. In order to detect an incidence rate of 10% per person per 24 weeks with a relative precision of 20% (from 8 to 12%) for each age group at 90% confidence level, and considering a 15% rate of loss to follow up, a sample of 80 participants was targeted for recruitment in each age category.

### Statistical analysis

Data were analysed using STATA version 15 (StataCorp, Texas, USA). Descriptive statistics were used to summarize the data. Household socioeconomic status (SES) was calculated as a weighted sum of data on household possessions and utilities, using principal components analysis and the scores divided into quintiles. Elements included in the measure: ownership of radio, TV, bicycle, refrigerator, mobile phone, computer, bed net, and source of energy (i.e., binary variable scoring presence or absence of electricity/gas/solar). The incidence rate of first infections was computed for 24 weeks of follow up as the total number of first infections observed after enrolment divided by total person time at risk.

Person-time was censored for loss to follow up or identification of a first infection. Incidence rate for multiple infections was computed for the 24 weeks of follow-up as the total number of malaria infections developing after enrolment divided by the total person-time at risk. Twenty-eight days were censored off from time at risk after each treatment of malaria infection. Kaplan Meier survival curves were used to compare time to first infection between groups, and Cox regression analysis was used to explore factors associated with first incidence of malaria infection. Lastly, proportional incidence of infection was computed as the number of participants developing first *P. falciparum* infection divided by the total number of participants at the start of follow up for each age category.

## Results

**Fig. 2:**
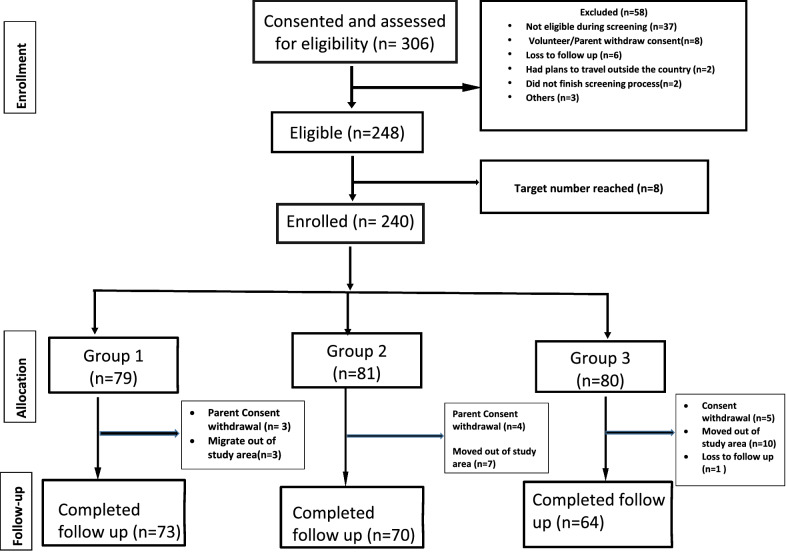
Consort diagram for the recruitment, group allocations and follow up of study participants in malaria incidence study on Bioko Island, Equatorial Guinea

Following the ordered sampling frame of participants identified from the BIMCP/NMCP household database who lived in the selected study areas (see “Methods”), 545 were contacted, and 306 expressed an interest to participate. After providing informed consent and undergoing screening, 66 were excluded: 37 did not meet the inclusion and exclusion criteria, 8 withdrew consent, 8 were not enrolled because the target number for recruitment was reached, 6 were lost to follow up, 2 did not finish the screening process, 2 had plans to travel outside the country, 1 left the study area, 1 did not complete AL pre-treatment and 1 had malaria by TBS 14 days after presumptive treatment (Fig. 2). 240 individuals completed pre-treatment, had a negative TBS thereafter and were enrolled. Screening and pre-treatment extended from 3rd of December 2018 to 15th of March 2019. One participant from the youngest group became 5 years old by the start of follow up and hence was re-categorized into the middle age group, leaving 79, 81 and 80 participants in groups 1 (6–59 months), 2 (5–17 years) and 3 (18–45 years), respectively, at the start of surveillance 14 days after AL.

The median age in years of those enrolled was 9.80 with range (IQR) from 1.02 to 42.51 (Table 1). The median ages and range for those in groups 1–3 were 2.65 (1.02, 4.99), 9.67 (5.01, 17.66) and 29.58 (18.3, 42.5) years, respectively. There were no infants recruited in Group 1 (also see Table 1). Overall, similar numbers of males and females were enrolled (ratio 0.91:1). The numbers of participants from the selected administrative areas of the city were well-balanced: Area I: 53; Area II: 63; Area III: 59; Area IV: 65. The distribution of wealth categorizes also appeared balanced between each age category (Table 1).

**Table 1:** Characteristics of 240 participants enrolled in malaria incidence study on Bioko Island, Equatorial Guinea (number (%))

Variable	Total	Age groups		
		6 to 59 months n = 79	5 to 17 years n = 81	18 to 45 years n = 80
Median age in years (range)	9.80 (1.02, 42.51)	2.65 (1.02, 4.9)	9.67 (5.01, 17.66)	29.58 (18.3, 42.5)
Sex				
Male	114 (47.5)	37 (46.8)	40 (49.4)	37 (46.3)
Female	126 (52.5)	42 (53.2)	41 (50.6)	43 (53.8)
Area				
Area I	53 (22.1)	18 (22.8)	18 (22.2)	17 (21.3)
Area II	63 (26.3)	27 (34.2)	21 (25.9)	15 (18.8)
Area III	59 (24.6)	10 (12.7)	19 (23.5)	30 (37.5)
Area IV	65 (27.1)	24 (30.4)	23 (28.4)	18 (22.5)
Household SES				
Lower	68 (28.3)	17 (21.5)	23 (28.4)	28 (35)
Upper lower	31 (12.9)	14 (17.7)	07 (8.6)	10 (12.5)
Middle	51 (21.3)	25 (31.6)	14 (17.3)	12 (15.0)
Upper middle	43 (17.9)	13 (16.5)	16 (19.8)	14 (17.5)
Upper	47 (19.6)	10 (12.7)	21 (25.9)	16 (20.0)
Use bed net as vector control	239 (99.6)	79 (100)	80 (98.8)	80 (100)
Clear vegetation around the house	185 (77.1)	61 (77.2)	59 (72.8)	65 (81.3)
Drying standing water	157 (65.4)	59 (74.7)	54 (66.7)	44 (55.0)
TBS positive before enrollment^b^	15 (6.3)	2 (2.5)	9 (11.1)	4 (5)
Haemoglobin level (mean ± SD)				
Male	11.9 ± (1.7)	10.7 ± (0.9)	11.4 ± (1.3)	13.6 ± (1.4)
Female	10.9 ± (1.3)	10.6 ± (1.2)	11.0 ± (1.3)	11.1 ± (1.3)
Creatinine level in mg/dL (median, range)^a^	0.47 (0.2, 2.04)	0.29 (0.20, 0.49)	0.49 (0.27, 0.80)	0.76 (0.23, 2.04)
Glucose level in mg/dL (median with range)	86 (61, 154)	88 (63, 154)	86 (62, 134)	84 (61, 147)

All values are number (%), unless specified otherwise

^a^N = 239, one subject in group 2 lacks creatinine results

^b^Malaria positive at screening or between screening and enrollment (within 2 months before enrolled into the study; however all participants were given presumptive treatment and retested before the day of initiation of the study and were negative by TBS before entering surveillance. Retrospective qPCR confirmed negative results in all included study subjects 14 days after AL treatment

Clinical parameters collected at screening for those enrolled in the three age categorizes attested to good health.

Surveillance began 14 days after completing AL administration, and extended for 24 weeks. The period of surveillance for the first participants began on 28th January 2019 and ended on 15th July 2019, and for the last participants began on 2nd April 2019 and ended on 12th September 2019. During the follow up 33 participants dropped out of the study (13.8% with 95% confidence intervals (CI) 10.0, 18.8), 6 participants in group 1, 11 participants in group 2, and 16 participants in group 3 (Fig. 2). The reasons for drop-out were: 20 migrated outside study area, 12 withdrew consent (3 in group 1, 4 in group 2 and 5 in group 3) and 1 was lost to follow-up.

There were 58 malaria infection events, 47 first events and 11 secondary events, of which 9 were second episodes and 2 were third episodes. All infection events positive by TBSs were confirmed as positive for Pf by retrospective qPCR. The results of the comparison between the different malaria diagnostics tests performed, including TBS, RDT and qPCR, will be presented elsewhere. Five of the second episodes and all third episodes were in participants from Area IV the most rural study area. Four of the second episodes were among participants aged 5–17 years and 3 were in those aged 18–45 years. All individuals with third episodes were aged 5–17 years.

The overall incidence rate throughout the study period was 0.25 (95% CI 0.19, 0.32) per person per 24 weeks follow-up. The overall incidence of first infection was 0.23 (95% CI 0.17, 0.30) per person per 24 weeks. The observed incidence rate for first infections was similar in the three age categories: 0.22 (95% CI 0.13, 0.36), 0.26 (95% CI 0.17, 0.42) and 0.20 (95% CI 0.12, 0.34) infections per 24 weeks in groups 1, 2 and 3, respectively (Table 2). First infection rates using a re-categorization of age ranges separating out 6–23-month-olds and, for those age 2 years and above, using the adjusted age ranges planned for the upcoming large trial, showed similar results: 0.15 (95% CI 0.05, 0.46) for 6–23-month-olds, 0.22 (95% CI 0.15, 0.34) for 2–12-year-olds, and 0.25 (95% CI 0.16, 0.38) for 13–45-year-olds.

**Table 2:** Incidence rate of malaria infection by TBS among participants enrolled in malaria incidence study on Bioko Island, Equatorial Guinea

	n	Events	Proportion positive (95% CI)	Incidence rate^a^ ( 95% CI)
All infections				
Total	240	58	0.24 (0.19, 0.30)	0.25 (0.19, 0.32)
First infections				
Total	240	47	0.19 (0.15, 0.25)	0.23 (0.17, 0.30)
Age group				
6 to 59 months	79	15	0.19 (0.12, 0.29)	0.22 (0.13, 0.36)
5 to 17 years	81	18	0.22 (0.14, 0.33)	0.26 (0.17, 0.42)
18 to 45 years	80	14	0.18 (0.11, 0.28)	0.20 (0.12, 0.34)
Sex				
Male	114	29	0.25 (0.18, 0.34)	0.31 (0.22, 0.45)
Female	126	18	0.14 (0.09, 0.22)	0.16 (0.10, 0.25)
Area				
Area I	53	7	0.13 (0.06, 0.26)	0.14 (0.07, 0.30)
Area II	63	7	0.11 (0.05, 0.22)	0.12 (0.06, 0.25)
Area III	59	13	0.22 (0.13, 0.35)	0.26 (0.15, 0.45)
Area IV	65	20	0.31 (0.21, 0.43)	0.39 (0.25, 0.61)
TBS positive before enrollment^b^				
Yes	15	6	0.4 (0.17, 0.68)	0.51 (0.23, 1.12)
No	225	41	0.18(0.14, 0.24)	0.21 (0.15, 0.29)
Household SES				
Lower	68	14	0.21 (0.12, 0.32)	0.25 (0.15, 0.43)
Upper lower	31	12	0.39 (0.23, 0.58)	0.46 (0.26, 0.82)
Middle	51	9	0.18 (0.09, 0.31)	0.20 (0.10, 0.38)
Upper middle	43	9	0.21 (0.11, 0.36)	0.26 (0.14, 0.50)
Upper	47	3	0.06 (0.02, 0.19)	0.07 (0.02, 0.20)
Travel^c^				
Yes	18	5	0.28 (0.11, 0.55)	0.32 (0.13, 0.78)
No	221	42	0.19 (0.14, 0.25)	0.22 (0.16, 0.3)

^a^Rate per 24 weeks

^b^Malaria positive by [TBS at screening or between screening and enrollment (within 2 months before enrolled into the study), however all participants were given presumptive treatment and retested before the day of initiation of the study

^c^Yes, if a person traveled at least once at any time point during the study

Incidence of infection appeared to be greater in: (1) males, 0.31 (95% CI 0.22, 0.45) versus females, 0.16 (95%CI 0.10, 0.25), the trend observed in all areas, and (2) those living in Areas III, 0.26 (95% CI 0.15,0.45) and IV, 0.39 (95% CI 0.25, 0.61) compared to those in Areas I, 0.14 (95% CI 0.07, 0.30) and II, 0.12 (95%CI 0.06, 0.25). Incidence was lowest in the highest socioeconomic group (Table 2).

**Fig. 3:**
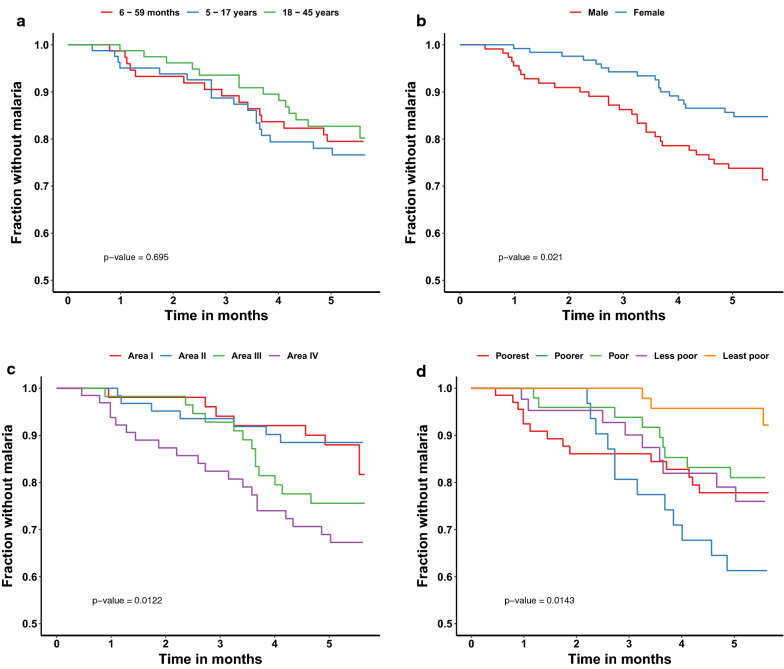
Survival curves comparing time to first malaria infection over the 6 months of malaria incidence study follow-up on Bioko Island, Equatorial Guinea. **A** Incidence of first malaria infection by age group; **B** incidence of first malaria infection by gender; **C** incidence of first malaria infection by area; **D** incidence of first malaria infection by quantile of social economic status of the household

The Kaplan Meier estimator showed trends toward significant differences for the chosen parameters (Fig. 3). The use of ITNs was universal and there were no differences related to the use of malaria control interventions during the study. None of the malaria infections were preceded by history of travel in the previous 2 weeks, indicating mainly local transmission. However, there were 47 participants who traveled at least once in the 24 weeks of follow up, and these individuals appeared to have a higher incidence rate (32%) than those who did not travel (22%) (Table 2). Multivariate assessment of the important factors predicting infection showed that study area, gender, being malaria positive at screening, and socio-economic status independently predicted the observed incidence of first malaria infection (Table 3).

**Table 3:** Factors associated with first malaria infection among participants enrolled in malaria incidence study on Bioko Island, Equatorial Guinea (Cox regression, N = 240)

Variable	Crude hazard ratio (95% CI)	Adjusted hazard ratio (95% CI)^a^
Age group		
6 to 59 months	1	1
5 to 17 years	1.21 (0.61, 2.41)	1.31 (0.63, 2.76)
18 to 45 years	0.9 (0.43, 1.86)	1.09 (0.51, 2.33)
Sex		
Male	1	1
Female	0.5 (0.28, 0.91)*	0.39 (0.21, 0.72)*
Area		
Area I	1	1
Area II	0.83 (0.29, 2.37)	1.17 (0.4, 3.44)
Area III	1.84 (0.73, 4.60)	1.85 (0.71, 4.81)
Area IV	2.78 (1.17, 6.57)*	3.63 (1.5, 8.8)*
TBS positive before enrollment^b^		
Yes	1	1
No	0.41 (0.17, 0.97)*	0.25 (0.1, 0.68)*
Household SES		
Lower	1	1
Lower middle	1.82 (0.84, 3.94)	3.35 (1.46, 7.68)*
Middle	0.76 (0.33, 1.76)	1.06 (0.45, 2.52)
Upper middle	1.03 (0.44, 2.37)	1.56 (0.66, 3.69)
Upper	0.25 (0.07, 0.87)*	0.31 (0.09, 1.14)

^*^Wald test p value < 0.05

^a^Adjusted for all variables in the table

^b^Malaria positive at screening or between screening and enrollment (within 2 months before enrolled into the study); however all participants were given presumptive treatment and retested before the day of initiation of the study

There were 31 cases of malaria with symptoms; these were 76% (n = 13), 42% (n = 10) and 47% (n = 8) of the total cases of malaria recorded in groups 1, 2 and 3 respectively. Of these, 27 were first cases of malaria with symptoms. The incidence rate for all cases of malaria with symptoms was 0.14 (95% CI 0.09, 0.19) and for first malaria with symptoms was 0.13 (95% CI 0.09, 0.19). The incidence rates of first cases of malaria with symptoms in groups 1, 2, 3 were 0.16 (95% CI 0.09, 0.30), 0.10 (95% CI 0.05, 0.22), 0.11 (95% CI 0.06, 0.22), respectively. Younger children and those living in Area IV tended to have more clinical episodes, but the numbers were small and the differences between areas were not statistically significant (Table 4). The incidence numbers for first malaria with symptoms using the adjusted age ranges consistent with the upcoming large trial were 0.10 (95% CI 0.03, 0.40) for 6–23-month-olds, 0.13 (95% CI 0.08, 0.23) for 2–12-year-olds, and 0.14 (95% CI 0.08, 0.24) for 13–45-year-olds.

**Table 4:** Incidence rate of malaria with symptoms by TBS among participants enrolled in malaria incidence study on Bioko Island, Equatorial Guinea

	n	Events	Proportion positive (95% CI)	Incidence rate^a^ (95% CI)
All clinical infections				
Total	240	31	0.13 (0.09, 0.18)	0.14 (0.09, 0.19)
First clinical infections				
Total	240	27	0.11 (0.08, 0.16)	0.13 (0.09, 0.19)
Age group				
6 to 59 months	79	12	0.15 (0.09, 0.25)	0.16 (0.09, 0.30)
5 to 17 years	81	7	0.09 (0.04, 0.17)	0.10 (0.05, 0.22)
18 to 45 years	80	8	0.10 (0.05, 0.19)	0.11 (0.06, 0.22)
Sex				
Male	114	14	0.12 (0.07, 0.19)	0.15 (0.09, 0.25)
Female	126	13	0.10 (0.06, 0.17)	0.11 (0.07, 0.20)
Area				
Area I	53	5	0.09 (0.04, 0.21)	0.10 (0.04, 0.24)
Area II	63	4	0.06 (0.02, 0.16)	0.06 (0.03, 0.18)
Area III	59	6	0.10 (0.04, 0.21)	0.12 (0.05, 0.27)
Area IV	65	12	0.18 (0.11, 0.30)	0.23 (0.13, 0.41)
TBS positive before enrollment^b^				
Yes	15	1	0.07(0.01, 0.41)	0.08 (0.01, 0.59)
No	225	26	0.12 (0.08, 0.16)	0.13 (0.09, 0.19)
Household SES2				
Lower	68	6	0.09 (0.04, 0.18)	0.10 (0.04, 0.24)
Upper lower	31	8	0.26(0.13, 0.45)	0.30 (0.15, 0.62)
Middle	51	6	0.12 (0.05, 0.24)	0.13 (0.06, 0.29)
Upper middle	43	5	0.12 (0.05, 0.26)	0.14 (0.06, 0.35)
Upper	47	2	0.04 (0.01, 0.16)	0.04 (0.01, 0.18)
Travel^c^				
Yes	18	3	0.17 (0.05, 0.51)	0.26 (0.08, 0.81)
No	221	24	0.11 (0.07, 0.15)	0.12 (0.08, 0.18)

^a^Rate per 24 weeks

^b^Malaria positive at screening or between screening and enrollment (within 2 months before enrolled into the study), however all participants were given presumptive treatment and retested before the day of initiation of the study

^c^Yes, if a person travels at least once at any time point during the study

The observed parasite density for symptomatic infections was higher than for asymptomatic infections with geometric mean parasites/µL of 1351.57 (95% CI 512.79, 3562.38) and 157.48 (95% CI 58.25, 425.72), respectively. The geometric mean parasite density for all cases of malaria with symptoms was 1898 (95% CI 431–8364), 357 (95% CI 88–1449) and 112 (95% CI 26–493) for age groups 6 to 59 months, 5 to 17 years and 18 to 45 years, respectively.

Common illnesses that occurred in the study participants included respiratory tract infections, gastroenteritis, diarrhoea, and skin infections. About 70% (95% CI 60, 79) of illnesses required medical attention during the 24 weeks of follow up, and the young age group had more events compared to the older age groups (Table 5).

**Table 5:** Other illnesses reported during the study among participants enrolled in malaria incidence study on Bioko Island, Equatorial Guinea (N = 240)

	n (%)	Age group		
		6 to 59 months n = 79	5 to 17 years n = 81	18 to 45 years n = 80
Total	108 (100)	63	17	28
Acute upper and lower respiratory infections	57 (52.78)	37	7	13
Gastroenteritis and diarrheal diseases	10 (9.26)	7	1	2
Infections of the skin and subcutaneous tissues	9 (8.33)	8	1	0
Injuries and open wound	7 (6.48)	1	3	3
Unspecified fever	6 (5.56)	4	0	2
Helminthiases	5 (4.63)	2	0	3
Allergic contact dermatitis	4 (3.70)	3	1	0
Bacterial infections	3 (2.78)	0	2	1
Others	7 (6.48)	1	2	4

## Discussion

The areas of Malabo surveyed in this study are characterized by ongoing moderate malaria transmission despite over 16 years of intensive malaria control interventions. As expected, there was variation in the incidence of infection in different locations of the city; the semi-rural locations of the study (Area III and Area IV) had significantly higher burden compared to the more urban locations. This heterogeneity indicates that malaria control interventions have variably impacted malaria prevalence and morbidity depending on the setting; importantly, however, nowhere have they interrupted transmission, a situation shared with many other settings in Africa [22–24]. The differences between locations were true for both males and females, with the former showing higher incidence rates in all locations.

Furthermore, significant local transmission remains despite the impact of the study itself, which, due to active surveillance and prompt treatment, could be expected to reduce the incidence of malaria infection in the participants beyond the effects of ongoing control measures, as has been observed in several sites in Africa [25, 26]. Hence, it is important that additional tools including vaccines be developed and implemented to fast track malaria control and elimination [8, 27].

The relatively similar incidences of both asymptomatic and symptomatic malaria among the age groups suggests that intensive control has shifted the age pattern of the risk of infection away from children compared to before the intervention [11], as seen elsewhere in Africa [28, 29]. This indicates that adults in Malabo have partially lost their acquired immunity, are at increased risk of malaria with or without symptoms, and will need additional protection when they visit areas of high transmission like mainland EG. The deployment of a malaria vaccine such as PfSPZ Vaccine aimed at protecting all age groups could be an effective additional tool for control and elimination of malaria in this setting.

Surprisingly, the results of this study indicate there was a significantly higher risk of malaria infection in males than in females, but no higher risk for other illness events including pneumonia and diarrhea. This sex difference in malaria infection, contrasting with similar studies in moderate transmission areas in Africa [30], extended to all age groups and thus, although partially explainable by gender-related sociocultural or occupational differences [22, 31], may reflect other factors as well. Animal and human studies suggest that gender-specific susceptibility to infection is related to hormonal differences [32, 33]. It remains to be seen if this difference will be confirmed in the large vaccine trial or more importantly if this translates into differences in protection or rates of adverse events related to vaccinations.

Travel has been documented to be an important contributing factor to the maintenance of local transmission in Malabo, and Bioko Island as a whole [34, 35]. However, no cases were categorized as travel-related though those individuals who had a history of travel tended to have more infections.

As expected, the incidence of malaria with symptoms was lower (13%) compared to asymptomatic infections (20%), likely due, at least in part, to intense follow up and prompt treatment [36] as the majority of the asymptomatic infections may have become symptomatic if untreated [37, 38]. Hence, the use of infection rather than infection plus symptoms as an end point for evaluation of malaria interventions is appropriate and safer [39]. Furthermore, the phase 3 evaluation of the pre-erythrocytic vaccine RTS,S/AS01 did not show any difference in the estimated efficacy between infection or clinical malaria with different parasitaemia density cut-offs.

Overall, there were few illnesses during follow up which is similar to what has been observed in other studies in Africa where good clinical care is provided as part of a study [25, 26]. As expected, most of the illness events were mild and concentrated in the younger children, with pneumonia and diarrhea being the common presentations [26].

This study was conducted to serve as a baseline for the conduct of a large safety and efficacy study of PfSPZ Vaccine that has so far shown promising results in EG and elsewhere [6, 7, 40]. The incidence across the age ranges of approximately 20% over 24 weeks and relatively good adherence to follow up of approximately 86% give confidence that a robust assessment of the safety and efficacy of PfSPZ Vaccine against natural infection can be conducted in Malabo. One caveat is that during the period during which the study was conducted, there was higher than average rainfall and an increase in health facility malaria cases of 44% across the island (unpublished BIMEP health Information system data); hence, the observed incidence may be on a higher side.

The study provided a good platform for capacity development, building the skills and confidence of the research team; the ability to conduct assessments of malaria incidence will be critical for tracking the success of future efforts to eliminate malaria from Bioko Island.

## Conclusion

Intensive malaria control efforts in Bioko Island have resulted in a large reduction in the prevalence of malaria; local transmission remains, however, and the risk of infection is now spread relatively evenly through all age groups, a finding which is consistent with areas that have changed from high to lower transmission areas. The remaining burden of malaria offers an opportunity to properly evaluate the efficacy and safety of PfSPZ Vaccine and establish the safety and efficacy data required to receive marketing authorization (licensure).

## Data Availability

The datasets used and/or analysed during the current study are available from the corresponding author on reasonable request.

## Abbreviations

AL: Artemether/lumefantrine
ALT: Alanine aminotransferase
AST: Aspartate aminotransferase
BIMCP: Bioko Island Malaria Control Programme
CHMI: Controlled human malaria infection
CI: Confidence interval
EDTA: Ethylenediaminetetraacetic acid
EG: Equatorial Guinea
EGMALEP: Equatorial Guinea Malaria Epidemiology Project
EGMOHSW: Equatorial Guinea Ministry of Health and Social Welfare
EKNZ: Ethics Committee of Northwestern and Central Switzerland
HIV: Human immunodeficiency virus
IHI IRB: Ifakara Health Institute Institutional Review Board
IHI: Ifakara Health Institute
IQR: Interquartile range
IRS: Insecticide residual spraying
ITN: Insecticide-treated nets
MCDI: Medical Care Development International
MUAC: Mid-upper arm circumference
MVPs: Mass vaccination programmes
NMCP: National Malaria Control Programme
PfSPZ: *Plasmodium falciparum* Sporozoite
PfSPZ-CVac: Chemo-attenuated sporozoite vaccine
RDT: Rapid diagnostic test
SHC: Sampaka Health Center
Swiss TPH: Swiss Tropical and Public Health Institute
TBS: Thick blood smear
VE: Vaccine efficacy
WHO: World Health Organization
